# Global research landscape and citation dynamics of oblique lumbar interbody fusion (OLIF): A bibliometric analysis of the 100 most influential articles

**DOI:** 10.1016/j.bas.2026.106043

**Published:** 2026-04-11

**Authors:** Badr Hafiz, Thamer Alsharif, Faisal Sukkar, Moaath Alghamdi, Ali Zaki Alhabib, Shuruq Obaid Alshammari, Saleh Baeesa

**Affiliations:** a Department of Neurosciences, King Faisal Specialist Hospital and Research Centre, Jeddah, Saudi Arabia; b Neurosurgery Department, King Abdulaziz Specialist Hospital, Taif, Saudi Arabia; c Department of Neurosurgery, Dr. Sulaiman Al Habib Medical Group Holding Company, Jeddah, Saudi Arabia; d Department of Neurosurgery, King Fahad General Hospital, Jeddah, Saudi Arabia; e Faculty of Medicine, Alfaisal University, Riyadh, Saudi Arabia

**Keywords:** Oblique lumbar interbody fusion (OLIF), Bibliometric analysis, Lumbar spine surgery, Spinal fusion, Degenerative lumbar disease, Minimally invasive spine surgery

## Abstract

**Introduction:**

Oblique lumbar interbody fusion (OLIF) is increasingly used as a minimally invasive anterolateral approach that enables interbody fusion, restoration of segmental alignment, and indirect neural decompression while avoiding limitations of posterior and transpsoas techniques. As OLIF-related publications have expanded rapidly, identifying the studies and research themes that have influenced the field is important.

**Methods:**

The Web of Science Core Collection was searched for OLIF-related publications. Records were screened and ranked by citation count following a PRISMA-based selection process. The 100 most cited articles published between 2003 and 2024 were included. Data regarding citation counts, publication year, journals, authors, institutions, countries, and keywords were extracted and analyzed using the Bibliometrix package in R. Core journals were determined using Bradford's law, and keyword relationships, thematic trends, and international collaboration networks were evaluated.

**Results:**

Publication activity was limited before 2013 but increased markedly thereafter, with influential studies appearing between 2017 and 2021. The included articles averaged approximately 78 citations per paper. Spine-focused journals, particularly World Neurosurgery and Spine, accounted for a substantial proportion of publications. Authors and institutions from Japan, China, and South Korea produced the greatest output, with collaboration involving the United States. Common research topics included indirect decompression, cage subsidence, fixation strategies, perioperative complications, and comparisons with TLIF and LLIF techniques.

**Conclusion:**

OLIF research has progressed from early feasibility reports toward studies addressing patient selection, surgical technique, and complication prevention. Future investigations should emphasize comparative study designs, standardized outcome reporting, multicenter collaboration, and longer clinical follow-up to strengthen evidence base.

## Highlights

• First focused bibliometric analysis of top 100 OLIF studies.
• Influential OLIF research peaked between 2017 and 2021.
• East Asia dominates authorship, institutions, and citations.
• Indirect decompression and subsidence drive citation impact.
• Evidence base remains largely observational and comparative.

## 1 Introduction

Low back pain remains the leading cause of years lived with disability worldwide and is strongly linked to degenerative lumbar disorders that frequently require surgical evaluation when conservative measures fail (Hartvigsen et al., 2018; GBD, 2019 Diseases and Injuries Collaborators, 2020). Over the past two decades, spine surgery, particularly lumbar fusion, has expanded substantially, driven by aging populations, increasing imaging utilization, evolving thresholds for operative management, and rapid diffusion of new implants and techniques (Deyo et al., 2010; Rajaee et al., 2012; Weinstein et al., 2007, 2008; Parajón et al., 2017; Tian et al., 2013). This growth has heightened the need for approaches that reliably address pain and disability while limiting perioperative morbidity, hospital stay, and access-related complications (Tian et al., 2013; Ozgur et al., 2006; Rodgers et al., 2011).

Conventional posterior approaches, such as PLIF and TLIF, provide direct decompression and stabilization; however, they are associated with paraspinal muscle injury, epidural scarring, blood loss, and risks to neural elements, especially in multilevel disease or revision settings (Ozgur et al., 2006; Rodgers et al., 2011). Minimally invasive (MIS) posterior fusion techniques have been developed to mitigate approach-related morbidity; systematic evaluations suggest that MIS strategies can reduce blood loss, length of stay, and short-term recovery burden while maintaining acceptable fusion and clinical outcomes in selected populations (Ozgur et al., 2006; Rodgers et al., 2011). Nonetheless, posterior MIS approaches traverse the posterior musculoligamentous envelope and can be limited by dura/nerve root manipulation and constrained endplate preparation angles, particularly in deformity, high-grade spondylolisthesis, or in patients with complex anatomy (Ozgur et al., 2006; Rodgers et al., 2011).

The lateral transpsoas corridor (LLIF/XLIF) extended MIS concepts by enabling larger interbody grafts and indirect decompression while minimizing posterior muscle disruption (Kirnaz et al., 2020; Arnold et al., 2012; Lai et al., 2023; Li et al., 2020a; Silvestre et al., 2012). However, the transpsoas trajectory can expose the lumbar plexus to traction or injury and may be less favorable at certain levels or in some anatomical variants, motivating the continued evolution of anterior and anterolateral corridors (Kirnaz et al., 2020; Arnold et al., 2012; Lai et al., 2023; Li et al., 2020a; Silvestre et al., 2012; Davis et al., 2014). The oblique corridor, classically described as an anterior-to-psoas pathway between the psoas and great vessels, aims to preserve MIS advantages while reducing psoas traversal and plexus-related risks, and it supports large cage placement and segmental lordosis restoration with indirect decompression in appropriately selected patients (Davis et al., 2014; Fujibayashi et al., 2015; Ohtori et al., 2015; Mehren et al., 2016; Hong and Soh, 2025; Woods et al., 2017; Sato et al., 2017; Molinares et al., 2016; Chung et al., 2017).

Therefore, oblique lumbar interbody fusion (OLIF) has emerged as a widely adopted approach for degenerative diseases, deformities, and revision contexts, with an increasing amount of comparative and outcome literature addressing radiographic correction, clinical improvement, fusion, and complication profiles (Kim et al., 2022; Park et al., 2024; Li et al., 2020b). As OLIF publications expand, clinicians and researchers require a clear, data-driven understanding of which studies have most shaped the field, how evidence themes have shifted over time, and where influential work is produced and disseminated (Li et al., 2020b; Hah and Kang, 2019; An et al., 2023). Bibliometric analysis provides structured quantitative tools to map research landscapes, identify citation classics, determine core journals (e.g., Bradford's law), evaluate author/institution networks, and visualize thematic evolution using established platforms and indicators (Donthu et al., 2021; Morante-Carballo et al., 2022; van Eck and Waltman, 2010). These methods have been increasingly applied in spine surgery to clarify high-impact knowledge domains and guide future research priorities (Donthu et al., 2021; Morante-Carballo et al., 2022; van Eck and Waltman, 2010).

Despite the growing adoption of OLIF and rapid publication acceleration since the mid-2010s, a focused bibliometric characterization of the most influential OLIF literature remains necessary to synthesize foundational evidence, highlight dominant research clusters, and expose underexplored areas that may be clinically important but under-cited. Accordingly, this study aimed to perform a bibliometric analysis of the top 100 most-cited OLIF-related articles to identify landmark publications, temporal trends, core journals, leading authors/institutions/countries, and thematic hotspots shaping OLIF research.

## 2 Methods

### 2.1 Study design

This study was designed as a bibliometric analysis of the most influential literature on OLIF. The aim was to identify, quantify, and map the characteristics of the top 100 most-cited publications, including trends in scientific production, citation impact, authorship patterns, institutional contributions, and thematic evolution within the field.

### 2.2 Data source

The Web of Science Core Collection, particularly the Science Citation Index Expanded (SCI-E), was used as the sole bibliographic database for data retrieval. This database was selected because it provides a standardized framework for citation indexing and ensures consistency in bibliometric indicators over time. Limiting the analysis to a single, well-established source reduced variability in citation metrics and improved the reliability and reproducibility of the bibliometric evaluation.

### 2.3 Search strategy and study selection

**Fig. 1:**
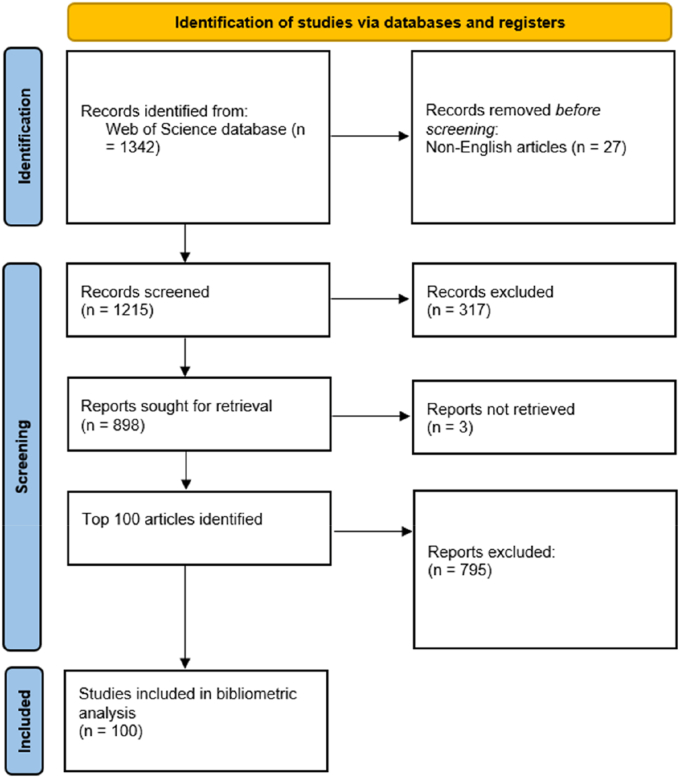
PRISMA flow diagram of study selection.

A comprehensive search of the Web of Science database was conducted to identify publications related to OLIF. Relevant keywords and indexing terms associated with oblique lateral interbody fusion were used to retrieve records. Filters were applied to refine the results, and duplicate records were removed. Titles and abstracts were screened to exclude irrelevant publications. The remaining records were ranked according to citation counts, and the top 100 most-cited articles were selected for inclusion. The study selection process is documented using a PRISMA flow diagram (Fig. 1).

### 2.4 Data extraction

Bibliographic data from the included articles were exported from Web of Science and organized for analysis. The extracted variables included publication year, authorship information, institutional affiliations, country of origin, journal source, total citations, citations per year, and normalized citation metrics. Additional information, such as keywords and abstracts, was collected to support the thematic and network analyses.

### 2.5 Bibliometric analysis and visualization

Bibliometric analyses were performed using the Bibliometrix package in R, supplemented by structured visualization workflows. The analysis included the assessment of annual scientific production, citation trends, most relevant authors, journals, affiliations, and countries, as well as the identification of the most-cited documents. Bradford's law was applied to determine core sources, and source dynamics over time were evaluated. Keyword frequency, co-occurrence networks, and thematic mapping were conducted to explore the conceptual structure of the field. Collaboration networks were generated to illustrate international research partnerships. All visualizations were produced using bibliometric mapping techniques to provide a comprehensive overview of research patterns and knowledge development in OLIF.

## 3 Results

After a comprehensive search of the Web of Science (WoS) database, relevant filters were applied to refine the retrieved records. After removing duplicates and screening titles and abstracts to exclude irrelevant publications, the top 100 most-cited articles related to OLIF were shortlisted for bibliometric analysis. The study selection process is illustrated in Fig. 1 using a PRISMA flow diagram. The main bibliometric characteristics of the included literature indicated a timespan from 2003 to 2024, with an annual growth rate of 0%. The average age of the documents was 7.48 years, and the mean number of citations per document was 77.77.

### 3.1 Temporal distribution of scientific production

**Fig. 2:**
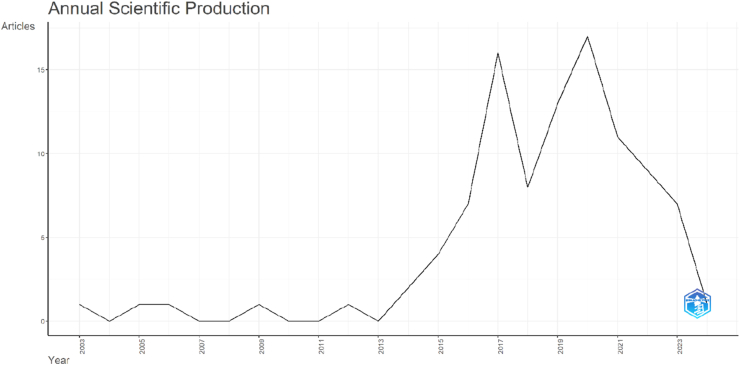
Time-series line graph showing the annual scientific production of the top 100 most-cited OLIF publications from 2003 to 2024.

A temporal analysis of the top 100 most-cited articles on OLIF demonstrated a gradual increase in scientific output over time (Fig. 2). The earliest included publication appeared in 2003, followed by a prolonged period of minimal productivity between 2003 and 2013, during which only isolated articles were published each year. From 2014 onward, a noticeable upward trend was observed, with annual publications rising from two articles in 2014 to seven in 2016. Scientific production peaked in 2017 with 16 articles and reached its highest level in 2020 with 17 articles, reflecting a period of intensified research interest and citation impact in OLIF. Although a slight decline was noted thereafter, the output remained relatively high, with 11 articles in 2021, nine in 2022, and seven in 2023. Only one article was recorded in 2024, likely due to the recency of publications and the time required to accumulate citations. Overall, the findings indicate that OLIF research gained substantial momentum after 2014, with the most influential studies concentrated between 2017 and 2021.

### 3.2 Annual citation trends

**Fig. 3:**
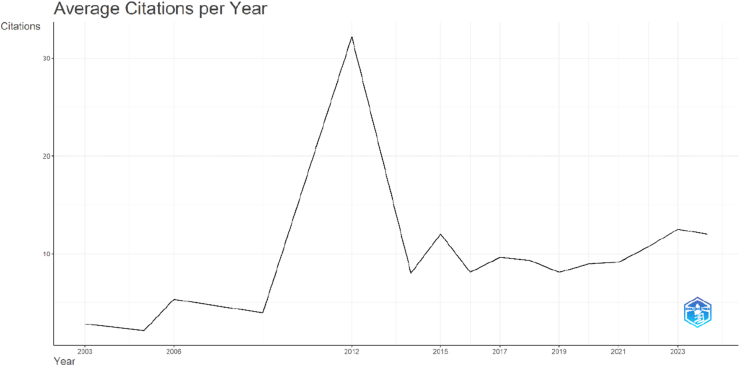
Time-series line graph illustrating the average citations per year of OLIF publications across the study period.

The annual distribution of citations demonstrated variability in the average citation impact of OLIF publications over time (Fig. 3). Early publications showed relatively low mean citations per year, with values of 2.79 in 2003, 2.14 in 2005, and 5.33 in 2006. A marked peak was observed in 2012, when the mean citations per year reached 32.20 despite only one article being published, indicating the high influence of that particular study. From 2014 onward, citation performance stabilized, with mean citations per year generally ranging between approximately 8 and 12. Articles published between 2015 and 2017 demonstrated consistent citation impact (12.02, 8.10, and 9.62, respectively), coinciding with the expansion of OLIF-related research. In subsequent years, citation rates remained steady, including 8.96 in 2020 and 9.12 in 2021, followed by a gradual increase to 10.71 in 2022 and 12.50 in 2023. Although 2024 showed a relatively high mean citation rate (12.00), this should be interpreted cautiously because of the limited number of citable years. Overall, the findings indicate that the OLIF literature has maintained a stable and sustained citation impact over the past decade, with occasional peaks driven by highly influential individual publications.

### 3.3 Most relevant sources

**Fig. 4:**
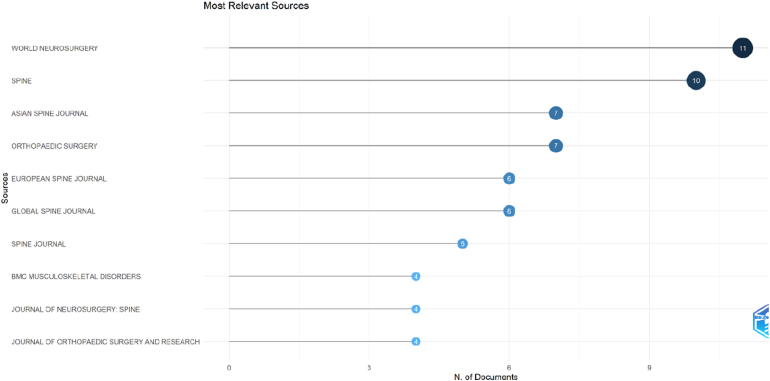
Dot plot showing the most relevant journals contributing to the top 100 most-cited OLIF articles based on the number of publications.

The distribution of the top 100 most-cited OLIF articles across journals identified several key publication venues (Fig. 4). *World Neurosurgery* emerged as the leading source, contributing 11 articles, followed closely by *Spine* with 10 publications. The *Asian Spine Journal* and *Orthopaedic Surgery* each published seven articles, while the *European Spine Journal* and *Global Spine Journal* contributed six articles each. Additional relevant sources included the *Spine Journal* (five articles), *BMC Musculoskeletal Disorders* (four articles), *Journal of Neurosurgery: Spine* (four articles), and *Journal of Orthopaedic Surgery and Research* (four articles). These findings highlight that OLIF research is predominantly concentrated in specialized spine and neurosurgical journals.

### 3.4 Bradford's law analysis

**Fig. 5:**
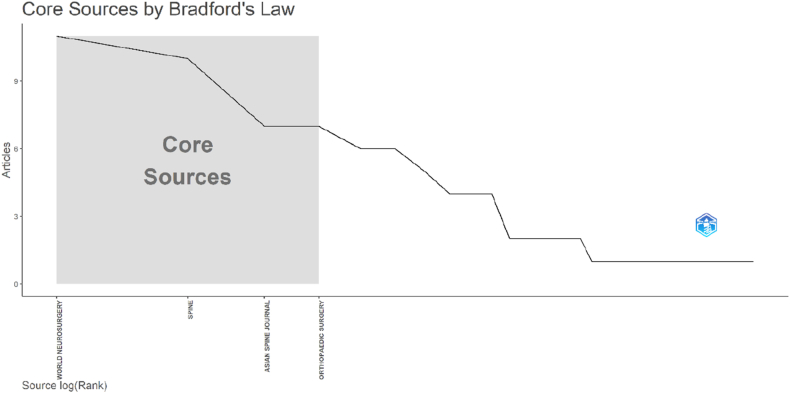
Bradford's law distribution plot identifying core journals contributing to the top 100 most-cited OLIF publications.

Bradford's law was applied to identify the core journals contributing to the OLIF literature (Fig. 5). A small group of highly productive journals formed the core zone, accounting for a substantial proportion of the most-cited articles, with *World Neurosurgery*, *Spine*, *Asian Spine Journal*, and *Orthopaedic Surgery* representing the primary core sources. The remaining journals were distributed across subsequent Bradford zones with progressively fewer contributions, demonstrating the typical bibliometric pattern of journal dispersion within a specialized research field.

### 3.5 Source dynamics over time

**Fig. 6:**
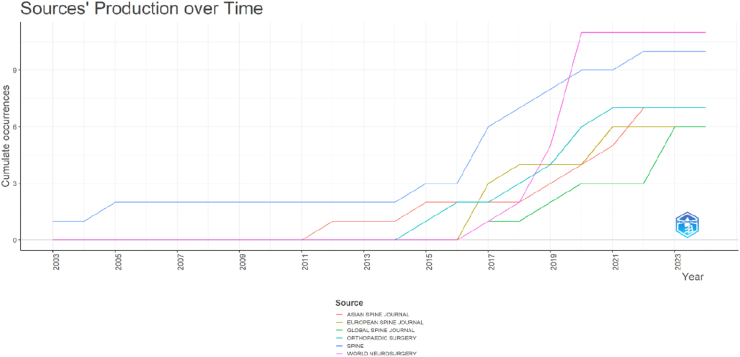
Multi-line cumulative time-series plot illustrating journal source dynamics and cumulative contributions of major journals to OLIF publications over time.

The analysis of source dynamics illustrated the temporal evolution of journal contributions to OLIF research (Fig. 6). Early publications were primarily dominated by *Spine*, which showed consistent contributions from the early years of OLIF research. From approximately 2015 onward, other journals, including *World Neurosurgery*, *Asian Spine Journal*, *European Spine Journal*, *Global Spine Journal*, and *Orthopaedic Surgery*, demonstrated increasing participation. *World Neurosurgery* exhibited the most notable growth in recent years, reflecting its expanding role as a major platform for OLIF-related research dissemination. Overall, these findings indicate a shift from concentration in a few established spine journals toward broader dissemination across multiple specialized surgical and musculoskeletal journals over time.

### 3.6 Most relevant authors

**Fig. 7:**
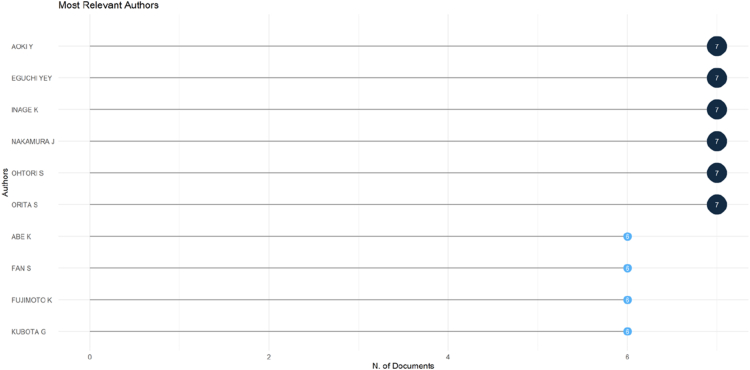
Dot plot showing the most productive authors in OLIF research based on the number of publications and fractionalized contributions.

The analysis of authorship identified several key contributors to the most-cited OLIF literature (Fig. 7). Aoki Y, Eguchi YEY, Inage K, Nakamura J, Ohtori S, and Orita S were the most productive authors, each contributing seven articles. Abe K, Fan S, Fujimoto K, and Kubota G followed, with six publications each. In terms of fractionalized contributions, Fan S demonstrated the highest proportional contribution (0.56), indicating substantial involvement as a primary or corresponding author across publications. Other leading authors, including Aoki Y, Eguchi YEY, Inage K, Nakamura J, Ohtori S, and Orita S, showed comparable fractionalized outputs (0.37), reflecting consistent collaborative participation within the OLIF research community. Overall, the authorship pattern suggests a concentrated group of researchers—predominantly from East Asia—driving high-impact OLIF research.

### 3.7 Most relevant affiliations

**Fig. 8:**
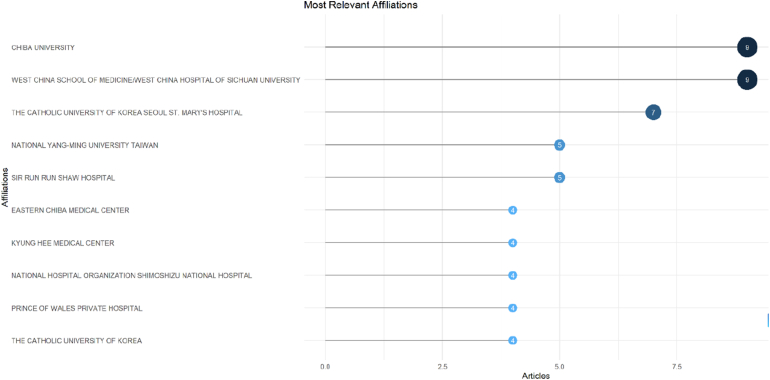
Dot plot showing the leading institutional affiliations contributing to the top 100 most-cited OLIF publications based on the number of articles.

Institutional analysis revealed that OLIF research is largely concentrated within a limited number of academic and clinical centers (Fig. 8). Chiba University and West China School of Medicine/West China Hospital of Sichuan University were the most productive affiliations, each contributing nine articles. The Catholic University of Korea Seoul St. Mary's Hospital followed with seven publications. Other notable institutions included National Yang-Ming University (Taiwan) and Sir Run Run Shaw Hospital (five articles each), while Eastern Chiba Medical Center, Kyung Hee Medical Center, National Hospital Organization Shimoshizu National Hospital, Prince of Wales Private Hospital, and The Catholic University of Korea each contributed four articles. These findings highlight the prominent role of leading Asian academic medical centers in advancing OLIF-related research.

### 3.8 Most cited countries

**Fig. 9:**
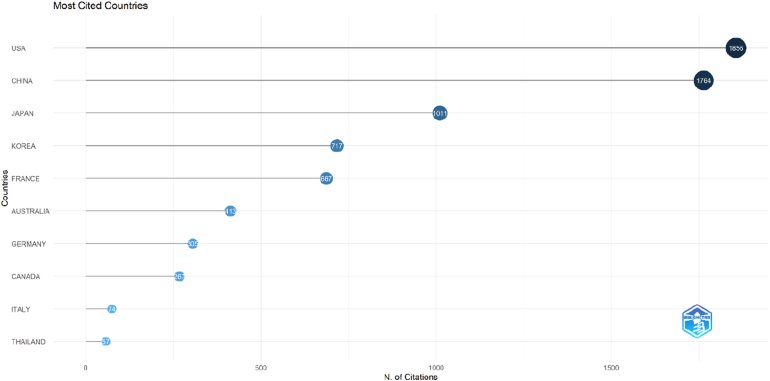
Dot plot illustrating the most-cited countries contributing to OLIF research based on total citation counts.

The geographic distribution of citations demonstrated that OLIF research impact is concentrated in a few leading countries (Fig. 9). The highest citation contributions were primarily from East Asian countries, particularly Japan, China, and South Korea, reflecting strong institutional and author-level productivity in these regions. Additional contributions were observed from countries such as the United States and other developed research systems, indicating international collaboration and global dissemination of OLIF knowledge. Overall, the results underscore Asia's central role, especially in Japan and China, in shaping the most influential OLIF literature and highlight the growing global engagement in this field.

### 3.9 Most cited documents

**Fig. 10:**
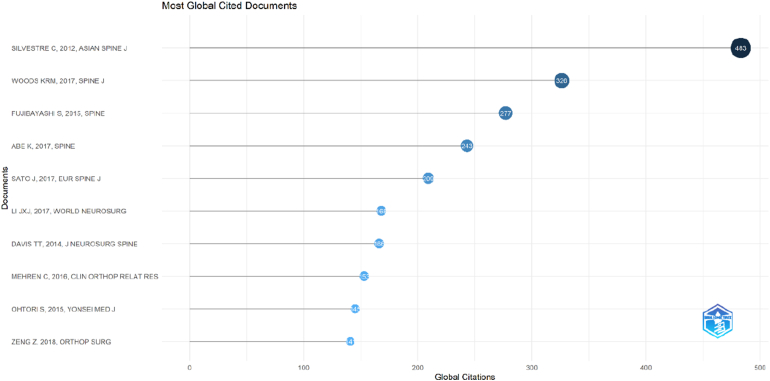
Dot plot ranking the top 10 most-cited OLIF articles based on total global citation counts.

The analysis of the most influential publications identified the top 10 most-cited articles in OLIF research (Fig. 10). The most-cited paper was published by Silvestre C in 2012 in *Asian Spine Journal*, accumulating 483 total citations and an average of 32.20 citations per year. This was followed by Woods KRM (2017, *Spine Journal*) with 326 citations and the highest citation rate per year (32.60), as well as the highest normalized citation impact (3.39). Other highly-cited studies included Fujibayashi S (2015, *Spine*) with 277 citations and Abe K (2017, *Spine*) with 243 citations. Sato J (2017, *European Spine Journal*) and Li JXJ (2017, *World Neurosurgery*) also demonstrated strong citation performance, with 209 and 168 citations, respectively. Additional influential publications were reported by Davis TT (2014, *Journal of Neurosurgery: Spine*), Mehren C (2016, *Clinical Orthopaedics and Related Research*), Ohtori S (2015, *Yonsei Medical Journal*), and Zeng Z (2018, *Orthopaedic Surgery*), each contributing substantially to the evidence base. Overall, these highly-cited articles represent foundational clinical and methodological studies that have shaped the development, adoption, and evaluation of OLIF techniques. Their high citation counts and sustained annual impact highlight their importance in guiding both clinical practice and subsequent research in this field.

### 3.10 Most frequent words

**Fig. 11:**
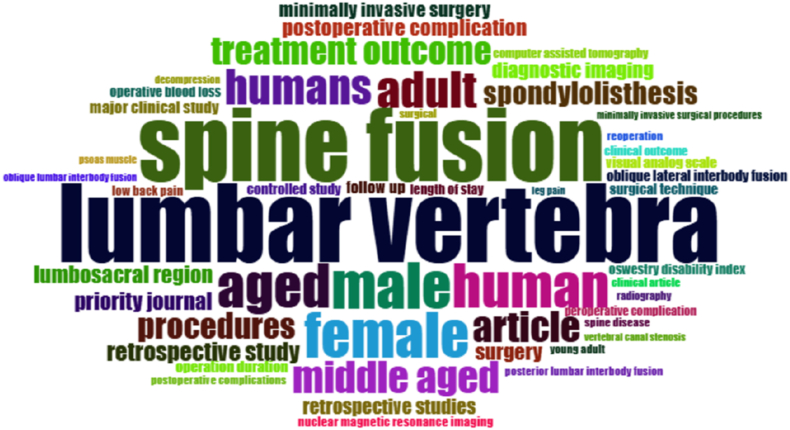
Word cloud visualization showing the most frequently occurring keywords in OLIF publications, with word size proportional to frequency.

The analysis of keyword frequency revealed the dominant thematic focus of the most-cited OLIF literature (Fig. 11). The word cloud demonstrated that the most frequently occurring terms were “lumbar vertebra,” “spine fusion,” and “humans,” indicating the central clinical emphasis of the research. Other prominent terms included “adult,” “aged,” “male,” and “female,” reflecting the primary patient populations studied. Clinical and procedural themes were also strongly represented, with frequent appearances of terms such as “treatment outcome,” “procedures,” “retrospective study,” “spondylolisthesis,” and “lumbosacral region.” Additionally, outcome-related and perioperative factors, including “postoperative complications,” “operation duration,” “length of stay,” and “visual analog scale, ”were commonly identified. These findings suggest that OLIF research predominantly focuses on clinical effectiveness, patient outcomes, and surgical indications.

### 3.11 Keyword co-occurrence network

**Fig. 12:**
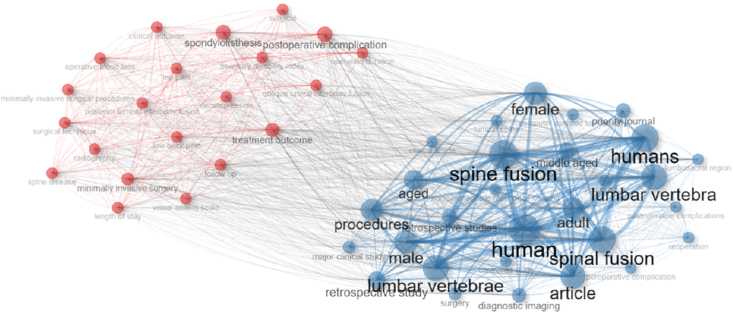
Keyword co-occurrence network graph illustrating thematic clusters and relationships among research topics in OLIF literature.

The co-occurrence network analysis illustrated the conceptual structure of OLIF research and the relationships among frequently used terms (Fig. 12). Two major thematic clusters were identified. The first cluster centered on clinical and surgical aspects, including “spine fusion,” “lumbar vertebra,” “procedures,” “retrospective studies,” and demographic descriptors such as “adult,” “aged,” and “humans.” The second cluster highlighted outcome-oriented and complication-related topics, including “treatment outcome,” “postoperative complications,” and imaging-related terms. The strong interconnections between these clusters indicate that OLIF research integrates surgical techniques with clinical outcomes and patient characteristics. Overall, the network demonstrates a well-developed and cohesive research landscape, with an emphasis on procedural evaluation, patient demographics, and postoperative results.

### 3.12 Thematic map of research topics

**Fig. 13:**
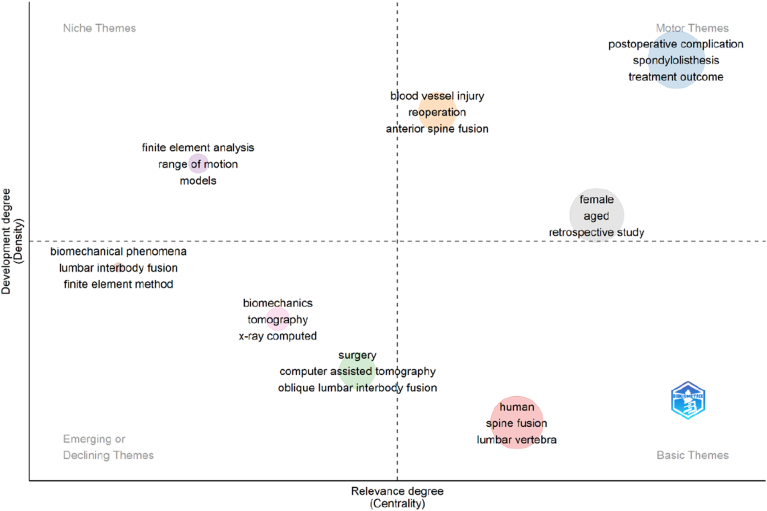
Thematic map of research themes in OLIF literature based on centrality and density.

The thematic map illustrates the conceptual structure and maturity of research themes in the OLIF literature based on centrality and density measures (Fig. 13). Motor themes, located in the upper-right quadrant, represent well-developed and highly relevant topics driving the field, including “postoperative complication,” “spondylolisthesis,” and “treatment outcome.” These themes reflect the strong clinical emphasis on surgical effectiveness and patient outcomes. The basic themes, positioned in the lower-right quadrant, include “human,” “spine fusion,” and “lumbar vertebra,” indicating fundamental and widely connected topics that form the core knowledge base of OLIF research. These themes are highly central but comparatively less developed, representing essential concepts underpinning the field. Niche themes, located in the upper-left quadrant, comprise specialized topics such as “finite element analysis,” “range of motion,” and modeling-related terms. These areas are well-developed but less central, suggesting focused methodological and biomechanical investigations. Emerging or declining themes, positioned in the lower-left quadrant, include topics such as “biomechanics,” “tomography,” and “finite element method,” as well as early procedural and imaging-related concepts. These themes may represent newly developing research directions or areas with decreasing attention over time. Overall, the thematic structure indicates that OLIF research is primarily driven by outcome-based clinical studies, supported by foundational surgical concepts, and evolving biomechanical and imaging methodologies.

### 3.13 Country collaboration network

**Fig. 14:**
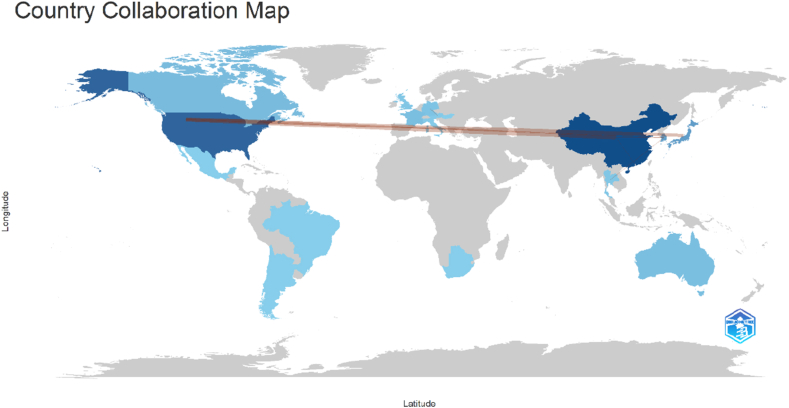
Geographic collaboration network map illustrating international research partnerships in OLIF research.

The global collaboration map illustrates international research partnerships in OLIF publications (Fig. 14). The analysis demonstrated that collaboration was predominantly concentrated among a limited number of highly productive countries, with strong connections observed between China, the United States, and Japan. These countries formed the central hubs of international cooperation, reflecting their leading roles in OLIF research output and citation impact. Additional collaborative links were identified with countries such as South Korea, Australia, and several European nations, indicating broader global engagement in the field. However, the intensity of collaboration outside the major hubs was comparatively lower, suggesting that OLIF research remains driven primarily by a few geographically concentrated research networks. Overall, the findings highlight the importance of transnational collaboration in advancing OLIF research, particularly between East Asia and North America, which appear to serve as the principal centers for knowledge production and dissemination in this domain.

## 4 Discussion

A rapidly expanding literature can paradoxically make a field harder to navigate: as new techniques mature and indications broaden, the signal becomes distributed across multiple subspecialty journals, countries, and clinical contexts. In this setting, bibliometric analysis becomes useful not as a substitute for clinical synthesis, but as a map of how knowledge has accumulated, which questions have dominated attention, where collaboration is concentrated, and which themes are emerging versus consolidating. For OLIF specifically, the technique's appeal, minimally invasive access, indirect decompression, alignment correction, and adaptability across pathologies has led to fast iteration in approach nuances, fixation strategies, and perioperative workflow, creating a strong rationale for quantitatively characterizing the intellectual structure of the field.

In this study, the top-cited OLIF literature spanning 2003–2024 showed a clear pattern of early foundational work followed by an acceleration in influential publications during the years in which the technique entered broader adoption. Across the top 100 most-cited articles, the mean citation burden and the average “age” of highly cited work suggested that the most influential contributions largely reflected the phase in which OLIF's feasibility, safety profile, and comparative value were being established, with later studies progressively focusing on refinements (L5–S1 feasibility, subsidence mitigation, navigation, single-position surgery, and complication avoidance). Country and institutional patterns further indicated that the center of gravity of influential output clustered in settings where both case volume and technical innovation pipelines are strong, with collaboration networks reflecting a mix of regional consolidation and selective international co-authorship. Keyword co-occurrence similarly pointed to two broad knowledge streams: one centered on surgical technique, instrumentation, complications, and biomechanical concerns, and another centered on indirect decompression, stenosis/spondylolisthesis phenotyping, and sagittal/coronal correction. The mechanism of indirect decompression was the most significant clinical theme identified in this analysis. The study by Fujibayashi et al. (2015), published in *Spine*, stands out as one of the most cited and influential papers in the entire dataset (Fujibayashi et al., 2015). This prospective study was transformative because it provided rigorous radiographic evidence that OLIF could achieve substantial decompression of the neural elements without entering the spinal canal. Fujibayashi and colleagues quantified the increase in the cross-sectional area (CSA) of the thecal sac and the neural foramen following the insertion of large OLIF cages, demonstrating that ligamentotaxis (tensioning of the annulus and longitudinal ligaments) could effectively “unbuckle” the ligamentum flavum and restore canal dimensions (Fujibayashi et al., 2015; Aleinik et al., 2021; Liu et al., 2023).

The impact of this finding cannot be overstated. Prior to this, the standard treatment for severe lumbar stenosis was direct posterior decompression (laminectomy), which destabilizes the spine and often necessitates fusion. The validation of indirect decompression via OLIF offered a paradigm shift: the ability to treat stenosis via a minimally invasive anterior approach that *adds* stability while decompressing the nerves (Park et al., 2022). The sustained citation of Fujibayashi's work, along with supporting studies by Sato et al., indicates that this mechanism is the central value proposition of OLIF (Sato et al., 2017). Sato further refined this by analyzing the limitations of indirect decompression and identifying factors, such as severe bony lateral recess stenosis or facet ankylosis, that might preclude a successful outcome (Park et al., 2022; Yingsakmongkol et al., 2022; Gajjar et al., 2021).

This thematic cluster also reveals a lively academic debate regarding the “limits” of indirect decompression. The top-cited papers are frequently referenced in discussions about when to add posterior direct decompression. The consensus in the literature appears to be that while OLIF is powerful, it is not a panacea for all types of stenosis. The high frequency of keywords like “foraminal height,” “disc height,” and “subsidence” in our co-occurrence network reflects the community's obsession with maintaining the distraction required for this indirect effect. If the cage subsides, decompression is lost; thus, the literature on indirect decompression is inextricably linked to the literature on cage biomechanics and subsidence (Kotheeranurak et al., 2023; Koo et al., 2025).

Comparative effectiveness research is a major driver of citations in this field. As a newer technique, OLIF had to prove its worth against established gold standards (TLIF) and its direct competitor (XLIF/LLIF). Our bibliometric analysis identified a robust cluster of meta-analyses and comparative cohort studies that served as the evidence base for these comparisons (Woods et al., 2017; Quillo-Olvera et al., 2018). The comparison between OLIF and XLIF/LLIF is the most politically and clinically charged comparison in the literature. The primary marketing and clinical advantage of OLIF is the avoidance of lumbar plexus injury. The top-cited papers consistently highlight that OLIF is associated with a significantly lower rate of transient thigh pain, hip flexor weakness, and sensorimotor deficits compared to the transpsoas approach. This neurological safety profile is the single most cited reason for adopting OLIF over XLIF in the reviewed literature. However, the literature also presents the “cost” of this safety: an increased risk of vascular injury. Comparative papers are often cited to weigh these risks—neurological (XLIF) versus vascular (OLIF)—allowing surgeons to tailor the approach to the patient's specific anatomy (Hong and Soh, 2025; Mobbs et al., 2015; Wong et al., 2024). The comparison with TLIF focuses on perioperative metrics and sagittal alignment. High-impact literature, such as the meta-analyses by Xiao et al. and others, consistently demonstrates that while fusion rates are comparable, OLIF offers superior restoration of disc height and lumbar lordosis with significantly less blood loss. The ability of OLIF to place a large, wide cage on the apophyseal ring contrasts favorably with the smaller “bullet” cages used in TLIF, which are more prone to subsidence and offer less lordotic correction. These studies are crucial for justifying OLIF in the treatment of degenerative spondylolisthesis and adult spinal deformities (ASD), where sagittal correction is a primary surgical goal (Xiao et al., 2024).

Biomechanical studies, particularly finite element analysis (FEA), have featured prominently in the top 100 papers, addressing whether standalone OLIF provides adequate stability. High-impact literature indicates that standalone OLIF provides good stabilization in flexion/extension and lateral bending but is inferior to constructs with posterior fixation, especially in rotation (Cai et al., 2022; Hao et al., 2023). While bilateral pedicle screws (BPS) remain the stability gold standard, less invasive options, such as cortical bone trajectory screws or lateral plate fixation, are being explored. Biomechanical evidence suggests that standalone OLIF may suffice for single-level collapse with good bone quality; however, instability, deformity, or osteoporosis require posterior fixation. This has established the “hybrid” approach (OLIF + percutaneous screws) as the standard procedure.

A key finding of this bibliometric analysis is the dominance of East Asian countries, China, Japan, and South Korea in high-impact OLIF research, unlike other spinal surgery areas, where the US and Europe lead. Institutions, such as Chiba University, West China Hospital, and The Catholic University of Korea, are the main knowledge creators. This “Asian Wave” may be attributed to favorable anatomical factors in Asian populations for OLIF procedures and healthcare systems with high-volume academic centers that enable large patient studies. The rapid adoption of MIS techniques has generated substantial datasets for comprehensive studies. The prominence of the Asian Spine Journal and World Neurosurgery in core sources reinforces this trend. This geographic concentration means that standard outcomes and anatomical norms are largely based on Asian cohorts, requiring Western surgeons to consider potential differences in body mass index and psoas morphology in their populations (Lin et al., 2022; Zhu et al., 2022; Wang et al., 2023).

The limitations of this study should be interpreted in the context of bibliometric methodology and the evolving nature of OLIF research. Because citation accrual is time-dependent, more recent high-quality studies may be underrepresented despite their clinical relevance, whereas older foundational work benefits from longer exposure. In addition, our analysis was restricted to the Web of Science Core Collection, which may have excluded relevant studies indexed exclusively in other databases such as Scopus or PubMed Central, potentially introducing selection bias. Database indexing practices may further limit the inclusion of regional journals, non-English publications, or articles not optimally classified under OLIF-related terms.

Citation-based ranking is also inherently imperfect, as citation counts may be influenced by factors unrelated to scientific quality, including self-citation practices and citations driven by controversy or academic debate rather than methodological rigor. Furthermore, the selection of the top 100 most-cited articles represents an arbitrary threshold and may have excluded other influential studies that fall just below this cutoff.

Finally, bibliometric analysis does not include formal assessment of methodological quality or risk of bias of the included studies. As such, citation frequency should not be interpreted as a surrogate for level of evidence or clinical superiority, but rather as an indicator of academic influence that must be considered alongside study design, bias risk, and clinical heterogeneity.

The strengths of this work include its focused evaluation of the most influential OLIF literature over a long-term horizon, enabling a structured view of how the field's intellectual priorities have evolved from feasibility and safety toward refinement, patient selection, and workflow optimization. By integrating performance indicators with thematic mapping, the analysis clarifies not only “who and where” influential work is produced, but also “what” conceptual clusters define the field. This combination can help clinicians, trainees, and researchers efficiently identify core evidence, understand the dominant questions shaping current practice, and recognize the sources that most strongly anchor current OLIF discourse.

Future research should prioritize evidence that closes the gap between influence and certainty. Clinically, the next phase of OLIF research should emphasize higher-quality comparative designs, standardized definitions for indirect decompression success/failure, and prospective capture of complications that are corridor- and level-specific—especially at L5–S1. Methodologically, multicenter registries and harmonized outcome sets would reduce heterogeneity and improve the credibility of pooled analyses. Technically, ongoing innovation around navigation, single-position workflows, and fixation personalization should be paired with robust radiation reporting, cost-effectiveness evaluations, and long-term follow-up that includes fusion assessment and ASD incidence. From a research strategy standpoint, bibliometrics can be repeated periodically to monitor whether emerging themes (bone-quality-guided planning, subsidence prevention, minimally disruptive revision strategies, and L5–S1 corridor optimization) translate into higher-level evidence and, ultimately, improved patient-centered outcomes.

## 5 Conclusion

This bibliometric analysis of the 100 most-cited publications on OLIF provides a structured overview of the intellectual architecture, temporal evolution, and thematic priorities that shape the field. Influential research accelerated after 2014, with peak productivity observed between 2017 and 2021. Citation impact was concentrated within a limited number of spine-focused journals and high-volume academic institutions, particularly in East Asia and the United States. The dominant research themes centered on clinical outcomes, indirect decompression, degenerative lumbar pathology, fixation strategies, and complication profiles, reflecting a maturation trajectory from technical feasibility to optimization and risk mitigation. Comparative effectiveness studies and subsidence-focused investigations have emerged as key citation drivers in recent years.

## Declaration of interests

The authors declare that they have no known competing financial interests or personal relationships that could have appeared to influence the work reported in this paper. No external funding, grants, or industry support was received for the conduction of the study, analysis, or preparation of the manuscript. The authors have not received honoraria, consulting fees, stocks, or other forms of compensation from entities with an interest in the subject matter discussed. All authors had full access to the data and take responsibility for the integrity and accuracy of the analysis. The manuscript has not been published previously and is not under consideration elsewhere.
